# Determination of professional job burnout and temperament (Mizaj) from the viewpoint of Traditional Persian Medicine and work-related variables among Iranian dentists: a cross-sectional study

**DOI:** 10.1186/s40359-022-00803-x

**Published:** 2022-04-08

**Authors:** Fereshteh Noori, Seyed-Kazem Kazemeini, Fatemeh Owlia

**Affiliations:** 1 Dentist, School of Dentistry, Shahid Sadoughi University of Medical Sciences, Yazd, Iran; 2 Specialist of Traditional Persian Medicine, Shahid Sadoughi University of Medical University, Yazd, Iran; 3 Department of Oral and maxillofacial Medicine, Social determinants of oral health research center, School of Dentistry, Shahid Sadoughi University of Medical Sciences, Yazd, Iran

**Keywords:** Burnout, Maslach Burnout Inventory, Temperament, Dentists, Persian medicine

## Abstract

**Background:**

Dentists are prone to professional burnout due to the nature of their work but this phenomenon could be prevented. Professional burnout has a great impact on different aspects of human life. No study has been published on determination of professional job burnout and temperament (Mizaj) from the viewpoint of Traditional Persian Medicine (TPM). The current study intends to touch upon this issue for the first time.

**Methods:**

In order to conduct this Cross-Sectional study based on a randomized sampling method, 145 dentists completed the 22-item Maslach Burnout Inventory questionnaires (MBI), and the 20-item Salmannejad Mizaj questionnaire. The study started since February, 2020 and ended in August, 2020 in Yazd, Iran. A total of 120 valid questionnaires were collected, with a response rate of 82.76%. Data analysis was performed using descriptive statistics, mean and standard deviation, analytical tests (including student t-test, one-way analysis of variance) by Spss17 (Chicago, USA) software.

**Results:**

Overall, 8.3% of responders had high emotional exhaustion, while 65.8% and 33.3% had moderate depersonalization and reduced personal accomplishment, respectively. With respect to the results, dentists with cold and dry temperaments experienced a higher level of burnout in emotional exhaustion while dentists with warm and wet temperaments had a higher level of burnout in depersonalization and reduced personal accomplishment dimensions. There was an insignificant difference between age, gender, work experience, number of working days per week, number of patients per day with the dimensions of burnout. Pearson correlation coefficient indicated there was a positive correlation between avoidance job and emotional exhaustion (r = − 0.22, *p* = 0.016).

**Conclusion:**

Based on the findings, it may conclude there was no significant difference in professional burnout between different temperaments among dentists.

## Background

Burnout is a workplace problem by virtue of practice in an institute with a conflict of multiple objectives [1]. It has been depicted as an important professional and personal problem among persons in different sectors, as well as in healthcare and medicine [1–3]. Studies provided evidence that dentists encounter various professional stressful situations. Working with ever-changing technologies and methods of practice, prolonged sitting on the chair, great precision while the eyes are focused on distinct points and fine movements of the fingers, treating anxious patients, and having a heavy workload are known as the main causes of stress among dentists [4, 5]. Previous survey have indicated that dentistry is turning into fatigue-inducing profession [4]. In addition, there is evidence that burnout in dentists has a negative impact on patients' health [6]. Patients will have low satisfaction ratings when visiting healthcare workers who suffer from burnout [7]. Therefore, it is not surprising many investigators have recently turned to shed light on the level of burnout in dentists [8–10].

Maslach et al. were among the first researchers to describe this concept. They described burnout as a syndrome that includes three dimensions: emotional exhaustion, depersonalization, and personal accomplishment [11]. Emotional exhaustion is the key to measuring burnout and is a response to stress that brought about negative job trends and a loss of interest in people. The second dimension is defining depersonalization as a negative distance response, and behaviors towards persons who are users of the service or care; reduced personal accomplishment is a negative self-assessment during work, feeling worthless [12].

In spite of neglecting TPM in past decades, nowadays it would be recognized as the first line of treatment in various diseases [13]. Temperament (Mizaj) is the turning point of the physiopathology of diseases that is the result of the influence of the qualities of the four elements, namely warmth, coldness, dryness, and wetness on each other. In medicine, Mizaj means the innate power in the body that could control all aspects of the body [14]. Concerning the TPM theories, moods and mental states depend on their temperament, therefore, it can be found that behaviors and tendencies are influenced by different Mizaj [15, 16]. People can be warmer, colder, drier, or wetter than balanced. Mizaj also affects moods, interests, and even fertility power [17].

The present study has three main objectives. The first is to illustrate the level and frequency of professional burnout and work-related factors among dentists in Yazd. The second objective is to determine the frequency of different Mizaj among them. Finally, to the best of our knowledge, no study has been published on determination of professional job burnout and Mizaj from the viewpoint of TPM. The current study intends to touch upon this issue for the first time.

## Methods

### Sample size calculation and sampling method

The sample size formula was calculated according one proportion estimation (prevalence of burnout: 64.1%) [18] and the 11% differences with the 5% marginal error, which resulted in a sample size of 145 dentists. The list of eligible dentists was coded from 1 to 367 using the list of dentists in the Yazd medical council. The 145 eligible dentists were randomly selected using a random number table and evaluated. Then, their names, details, and telephone numbers were extracted. The research started since February, 2020 and ended in August, 2020. With a response rate of 82.76%, a total of 120 valid questionnaires were collected. Twenty-five participants were excluded due to response bias.

### Inclusion and exclusion criteria

With respect to inclusion criteria, participants must be between 26 and 60 years old and had at least two years of clinical work experience. Dentists with a history of any neurological or mental illness, physical or mental disability, taking corticosteroids, or immunosuppressive drugs did not fulfill the inclusion criteria. All of them filled out the HADS questionnaire for detecting symptoms of anxiety and depression. If participants filled each of the questionnaires incompletely, they would be excluded from the study.

### Ethical consideration

The study was carried out in accordance with the Declaration of Helsinki guidance of the World Medical Association and Good Clinical Practice recommendations. Dentists were personally visited and they were asked to write demographic characteristics and work-related data in the checklist. They were informed of its purpose and course, and of the possibility of withdrawing from the study. Informed consent for participation was obtained from all participants for inclusion before they entered the study. The anonymity and confidentiality of the participants were guaranteed. This study was approved by the "Ethics Committee in Research of Shahid Sadoughi University of Medical Sciences, Yazd" under the number IR.SSU.REC.1398.192.

### Measures

Basic demographic data were obtained from all participants including gender, age, work practice experience (in years), working days per week, number of patients seen per day, and avoidance or selective job. Involuntary (avoidance) or voluntarily (selective) job means the participants voluntarily select dentistry or they are forced to do it. A very detailed history of systemic diseases, taking medication and physical disability was taken.

### Professional burnout determination

Professional burnout level was evaluated by the Maslach Burnout Inventory questionnaires (MBI) questionnaire. The Persian version of the MBI questionnaire was filled out by participants [19]. It should be noted that there was no time limit for completing the questionnaire and therefore, it was tried to distribute and collect the questionnaires in a suitable situation. MBI questionnaire with Cronbach's alpha coefficient of 0.75 composed of 22 items of self-reported questions. It falls into 3 main dimensions, emotional exhaustion (EE), depersonalization (DP), and personal accomplishment (PA), with 9, 5, and 8 questions, respectively. Each question expressed the level of burnout with a limited range from zero (never) to 6 (always). Consistent with previous similar researchs [3, 8], the dentists were allocated into one of the three groups a high, moderate, or low level of burnout. Those with EE sum score of ≥ 27 were considered to have high EE level, 17–26 moderate, and 0–16 low level. Those with DP sum score of ≥ 13 were considered to have high DP level, 7–12 moderate and 0–6 low level. Those with PA score of ≤ 31 were considered to have low PA level, 32–38 moderate, and ≥ 39 high level.

### Mizaj determination

Salman-Nejad mizaj identification questionnaire contains 20 items that evaluate two categories of warmness/coldness and dry/ wet nature. It can be used for individuals aged 20 to 60 years old. The subjects rated their responses on a 0–5 scale. It is the first and most reliable and validated questionnaire with Cronbach's alpha coefficient equal to 0.77–0.80 [20].

### Data analysis

All of the analyses were conducted at a *p* < 0.05 level of significance using the SPSS statistical package (version 17.0; SPSS Inc., Chicago, IL, USA; 2012). Descriptive analyses were used to describe sample characteristics, sum score of Mizaj questionnaire, and subscale scores of MBI questionnair. Student t-tests and one-way ANOVA were used for data analysis.

## Results

Ultimately, 120 dentists (66 male and 54 female) who completely filled both questionnaires, constituted the samples of this study. The mean age ± SD of the samples was 35.2 ± 7.2 with a range of 28 to 60 years. Data analysis proved able that 66.7% of dentists had low emotional exhaustion, 65.8% had moderate depersonalization and 64.2% had a low personal accomplishment (Table 1).

**Table 1:** Burnout subscales scores of dentists based on MBI scales

Maslach burnout dimensions	High	Moderate	Low
	Number (percent)	Number (percent)	Number (percent)
Emotional exhaustion	10 (8.3%)	30 (25.0%)	80 (66.7%)
Depersonalization	9 (7.5%)	79 (65.8%)	32 (26.7%)
Personal accomplishment	3 (2.5%)	40 (33.3%)	77 (64.2%)

As can be seen from Table 2, the number and percentage of individuals in both genders and various Mizaj groups are presented. The most frequent Mizaj was related to warm and wet and the lowest frequency was attributed to temperate-temperate.

**Table 2:** Percentage of the frequency of different Mizajes in men and women

Different Mizaj	Male number (%)	Female number (%)	Total number (%)
Warm and wet	21 (31.8%)	13 (24.1%)	34 (28.3%)
Warm and dry	9 (13.6%)	7 (12.9%)	16 (13.3%)
Cold and dry	3 (4.5%)	6 (11.1%)	9 (7.5%)
Cold and wet	10 (15.5%)	6 (11.1%)	16 (13.3%)
Warm and temperate	4 (6.1%)	5 (9.3%)	9 (7.5%)
cold and temperate	5 (7.6%)	5 (9.3%)	10 (8.3%)
Temperate and dry	5 (7.6%)	4 (7.4%)	9 (7.5%)
Temperate and wet	9 (13.6%)	5 (9.3%)	14 (11.8%)
Temperate and Temperate	0 (0%)	3 (5.5%)	3 (2.5%)
Total	66 (100%)	54 (100%)	120 (100%)

As it is set out in Table 3, dentists with cold and dry Mizaj had a higher level of burnout in emotional exhaustion while dentists with warm and wet Mizaj had a higher level of burnout in depersonalization and personal accomplishment dimensions. Results of a one-way ANOVA indicated there was an overall insignificant difference between different Mizaj in terms of burnout dimensions (*p* value > 0.05).

**Table 3:** Comparison of Professional burnout in different Mizaj of participants

Different mizaj	Number	Emotional exhaustion	Depersonalization	Personal accomplishment
Warm and wet	34	14.70 ± 11.83	7.58 ± 3.03	30.88 ± 5.41
Warm and dry	16	8.37 ± 6.08	6.56 ± 2.92	30.81 ± 6.90
Cold and dry	9	16.77 ± 9.62	7.11 ± 2.26	30.11 ± 4.31
Cold and wet	16	14.06 ± 10.64	6.87 ± 2.27	28.87 ± 5.04
Temperate and wet	14	14.85 ± 1068	7.42 ± 1.86	29.92 ± 5.58
Temperate and dry	9	15.55 ± 9.70	6.55 ± 2.65	29.77 ± 5.60
Warm and temperate	9	13.11 ± 11.51	7.44 ± 4.03	28.22 ± 4.14
Cold and temperate	10	12.80 ± 9.69	7.30 ± 2.31	29.80 ± 4.73
Temperate and Temperate	3	10.0 ± 5.29	5.66 ± 1.52	30.66 ± 3.05
p-value		0.609	0.908	0.931

ANOVA

Table 4 provides an overview of the level of burnout in dentists based on gender and work-related variables. Both genders conveyed a similar burnout level in reduced personal accomplishment, emotional exhaustion, and depersonalization, respectively. Although the burnout level was higher in men, no statistically significant was seen between two genders. On the other hand, with enhancing the number of patients per day and the number of working days per week, although the rate of burnout in emotional exhaustion and depersonalization were generated, these differences were insignificant.

**Table 4:** Comparison of MBI dimensions in different mizaj in terms of related variables

Variables	Options	Number (%)	Emotional exhaustion Mean ± SD	Depersonalization Mean ± SD	Reduced personal accomplishment Mean ± SD
Age groups	< 35	69 (57.5%)	13.73 ± 11.18	7.27 ± 2.91	30.18 ± 5.60
	≥ 35	51 (42.5%)	13.45 ± 8.92	6.96 ± 2.35	29.88 ± 4.85
	*p* value		0.880	0.529	0.755
Gender	Female	54 (45%)	13.51 ± 9.96	6.77 ± 2.40	29.25 ± 5.36
	Male	66 (55%)	13.69 ± 10.55	7.43 ± 2.88	30.71 ± 5.15
	*p* value		0.925	0.181	0.134
Work practice experience (year)	< 7	61 (50.8%)	14.39 ± 11.71	7.36 ± 2.84	30.55 ± 4.92
	≥ 7	59 (49.1%)	12.81 ± 8.49	6.91 ± 2.52	29.54 ± 5.61
	*p* value		0.401	0.367	0.294
Working days per week	< 5	52 (43.3%)	12.77 ± 10.30	7.09 ± 2.09	30.49 ± 5.24
	5 ≤	67 (55.8%)	14.28 ± 10.23	7.17 ± 3.09	29.71 ± 5.31
	*p* value		0.425	0.865	0.427
Number of patients per day	3–4	25 (20.8%)	13.32 ± 9.62	7.04 ± 3.04	31.60 ± 4.08
	5 ≤	95 (79.1%)	13.69 ± 10.45	7.16 ± 2.60	29.65 ± 5.49
	*p* value		0.872	0.833	0.101
Selective or avoidance employment	Selective	94 (78.3%)	12.43 ± 10.07	7.05 ± 2.68	30.19 ± 4.95
	Avoidance	26 (21.6%)	17.88 ± 9.90	7.46 ± 2.71	29.57 ± 6.39
	*p* value		0.016*	0.495	0.601

T-test

*Significant

Dentists with more work practice experience experienced an insignificant diminished burnout in all of the dimensions of burnout.

The average burnout in the emotional exhaustion dimension was significantly higher in dentists with avoidance jobs (Table 4). Pearson correlation coefficient indicated there was a positive correlation between avoidance job and emotional exhaustion (r = − 0.22, P = 0.016).

## Discussion

Although the term “Mizaj” in TPM is mostly interpreted as “Temperament” in English, temperament is commonly used in psychology to introduce personality traits and mood. It appears that psychologists have borrowed this term. Of course, there seem to be some differences between temperament and Mizaj in TPM and in psychology [21].

According to the obtained results, low emotional exhaustion was the most common (66.7%), but moderate depersonalization and low personal accomplishment—also occurred frequently. The level of burnout in this research was significantly lower than in some studies among dentists [4, 22]. It may be related to the discrepancy in sample size, socioeconomic conditions, and various personal characteristics. These findings go in line with another study [23]. This similarity could be justified by the cultural affinity of the two Iranian societies. High rate of dentists willingly chose this job (78.3%) may play an important role in burnout reduction. Awareness of personal characteristics by Mizaj determination effectively facilitates the job selection process and improves their assumption about it [24].

On the basis of our results, the levels of the burnout dimensions did not tend to vary in relation to age. The values are barely distinguished from previous studies [25, 26]. However, other pieces of literatures mentioned a negative significant relationship between age and depersonalization and reduced personal accomplishment [18, 23]. In spite of an insignificant difference, dentists of older age (35 or more years aged), exhibited a lower level of burnout in all dimensions than the younger group. This finding is at variance with another research finding. Slabšinskien et al. indicated dentists of older age (40 or more years aged), had a meaningful lowered mean depersonalization sum score [4]. Having more working experience and professional skills with aging could introduce a lower level of work stress in this study.

No significant difference was recognized between gender and burnout. Even though this result differs considerably from previous studies [23, 25]. However, Eslamipour et al. proposed that the frequency and severity of emotional exhaustion and depersonalization were significantly higher in men [23]. While another researcher stated being female increased the emotional exhaustion sum score [4]. As far as our experience is concerned, this issue is associated with masculine temperament in female employees, this characteristic will be reinforced in dentistry especially noticeable in general dentists. Perhaps a different result would be obtained if this work was conducted in the specialized fields of dentistry such as pediatric. This field deal with subtle feminine moods and more noticeable gender differences [27].

On the basis of the results, there was no significant difference between the work experience and burnout. Although, dentists with work practice experience of less than 7 years have a higher burnout sum score than another group. This is in good agreement with previous results [25, 26]. Other researchers mentioned the direct relationship between work experience and dimensions of emotional exhaustion and personal accomplishment [4, 22]. Having more working experience and professional skills could introduce a lower level of work stress; therefore, job burnout may have diminished.

Lack of a significant difference between burnout dimensions and work-related variables such as working days per week, number of patients per day, and work experience in our study could suggest not all factors need to be weighed in future studies, separately.

The finding proposed a significant difference between the selective or avoidance job and emotional exhaustion. It may be concluded dentists who willingly chose their job significantly had lower emotional exhaustion. Regarding the past literature, the difference between job satisfaction and different dimensions of burnout was negative and significant [28, 29].

Attention to Mizaj could have an important role in job satisfaction. This work was similar to previous studies which evaluated different personality types with burnout [2, 30]. Abedi et al. determined the effect of different personality traits of managers on the job satisfaction of employees. They deducted the coefficients of warmth, coldness and dry and wet temperaments on job satisfaction were significant [31]. Another research found a positive association between psychological character type and burnout [32]. Salmannezhad et al. stated there is a positive correlation between happiness and warmness and a negative correlation between happiness and coldness of Mizaj [33]. On the other hand, Javadi sharif et al. mentioned there was a negative correlation between total job burnout and happiness [21]. Our finding somehow confirms this result. The most emotional exhaustion was related to people with cold and dry Mizaj.

Although great effort has gone into the field of Mizaj, less attention has been given to examining its correlation with burnout [34, 35]. On the strengths of this work, this is the first time to touch upon the professional burnout level of dentists in view of TPM. The study included standardized instruments that are validated measures for Mizaj and burnout. Our approach would lend itself well for use by further research.

Despite negative effects of burnout on job performance, family, and personal role, absenteeism from work, and early retirement due to burnout should not be neglected [24]. It is possible to minimize stressors by carefully considering the people, s Mizaj before entrance to job positions. It would be suggested that people should choose their job willingly to suffer less burnout in the future.

## Limitations

We are aware that our research suffers a number of limitations. The most important limitation lies in our response rate was unknown. It is vital to be aware of the predictive limitations of cross-sectional studies, and a causal relationship can be clarified by carrying out additional prospective research. There is clearly much room for further research in this respect.

## Conclusion

In conclusion, there were insignificant differences between age, gender, work experience, number of working days per week, number of patients per day with burnout. There was no significant difference in professional burnout between different Mizaj among dentists. The significance of the variable of avoidance or selective-employment focused on the crucial point that dentists with selective jobs, whether female or male, with each workload, will experience less professional burnout during working life.

## Data Availability

The datasets analyzed during the current study are available from the corresponding author on reasonable request.
